# Regulation of AKT Phosphorylation at Ser473 and Thr308 by Endoplasmic Reticulum Stress Modulates Substrate Specificity in a Severity Dependent Manner

**DOI:** 10.1371/journal.pone.0017894

**Published:** 2011-03-21

**Authors:** Hong Wa Yung, D. Stephen Charnock-Jones, Graham J. Burton

**Affiliations:** 1 Centre for Trophoblast Research, University of Cambridge, Cambridge, United Kingdom; 2 Department of Obstetrics and Gynaecology, University of Cambridge, Cambridge, United Kingdom; 3 Cambridge Comprehensive Biomedical Research Centre, National Institute for Health Research, Cambridge, United Kingdom; Cleveland Clinic, United States of America

## Abstract

Endoplasmic reticulum (ER) stress is a common factor in the pathophysiology of diverse human diseases that are characterised by contrasting cellular behaviours, from proliferation in cancer to apoptosis in neurodegenerative disorders. Coincidently, dysregulation of AKT/PKB activity, which is the central regulator of cell growth, proliferation and survival, is often associated with the same diseases. Here, we demonstrate that ER stress modulates AKT substrate specificity in a severity-dependent manner, as shown by phospho-specific antibodies against known AKT targets. ER stress also reduces both total and phosphorylated AKT in a severity-dependent manner, without affecting activity of the upstream kinase PDK1. Normalisation to total AKT revealed that under ER stress phosphorylation of Thr308 is suppressed while that of Ser473 is increased. ER stress induces GRP78, and siRNA-mediated knock-down of GRP78 enhances phosphorylation at Ser473 by 3.6 fold, but not at Thr308. Substrate specificity is again altered. An *in-situ* proximity ligation assay revealed a physical interaction between GRP78 and AKT at the plasma membrane of cells following induction of ER stress. Staining was weak in cells with normal nuclear morphology but stronger in those displaying rounded, condensed nuclei. Co-immunoprecipitation of GRP78 and P-AKT(Ser473) confirmed the immuno-complex consists of non-phosphorylated AKT (Ser473 and Thr308). The interaction is likely specific as AKT did not bind to all molecular chaperones, and GRP78 did not bind to p70 S6 kinase. These findings provide one mechanistic explanation for how ER stress contributes to human pathologies demonstrating contrasting cell fates via modulation of AKT signalling.

## Introduction

The endoplasmic reticulum (ER) stress has been postulated to play a causative role in a number of common human diseases such as cancer, diabetes, metabolic dysfunction, neurodegenerative diseases and pregnancy disorders. The ER is essential for the synthesis, maturation and export of secreted and membrane proteins including hormones, growth factors and membrane receptors. Any disturbance of ER homeostasis induced, for example, by nutrient deprivation, hypoxia, ischemia, inhibition of protein glycosylation or disulphide bond formation, and viral or bacterial infection, can result in excessive accumulation of misfolded or unfolded proteins in the ER lumen. This accumulation leads to ER stress, and triggers the unfolded protein response (UPR) [1]. To restore ER homeostasis, the UPR induces a number of protective mechanisms, including transient attenuation of protein translation, induction of molecular chaperones and folding enzymes, and increased degradation of misfolded proteins. If these adaptive responses fail to alleviate the stress, apoptotic pathways are activated to eliminate the damaged cells [2].

Among the various ER chaperones, glucose-regulated protein 78 (GRP78, also known as BiP), is the most abundant. GRP78 resides primarily in the ER lumen, or associated with the inner aspect of the ER membrane because of the ER retention motif, KDEL, at its carboxyl terminus. However, there is emerging evidence that GRP78 can localize to the plasma membrane under pathological conditions [3]. A recent publication from Zhang *et al.* demonstrated that ER stress promotes GRP78 localization on the cell surface in a severity-dependent manner [4]. In addition, GRP78 also exists in a cytosolic form, referred to as GRP78va. This variant results from alternative splicing within the intron between exon 1 and 2, thereby losing the ER-targeting signal peptide at the N-terminus. It has a molecular weight of about 62 kDa [5].

GRP78 is able to modify the function or activity of a variety of kinases/proteins through direct and indirect interactions upon stress or other stimuli. Client proteins include signalling kinases, Raf1 [6], proapoptotic proteins, caspase-7 [7] and BIK [8], and transcription factors, p53 [9]. The cellular importance of GRP78 is reflected by a study from Luo *et al.*, in which knock-out of the *Grp78* gene led to lethality at E3.5, indicating that GRP78 is essential for embryonic cell survival and growth [10].

The diseases associated with ER stress often exhibit abnormal AKT activity [11], [12]. AKT, a serine/threonine protein kinase, also known as protein kinase B and a member of AGC family, regulates a variety of cellular processes including survival, proliferation, protein translation and metabolism [13]. AKT contains a pleckstrin homology (PH) domain which binds to PIP_3_ (phosphatidylinositol (3,4,5)-trisphosphate, PtdIns(3,4,5)*P*_3_) in the plasma membrane with high affinity [14]. Once in correct position in the membrane, AKT can be phosphorylated by 3-phosphoinositide dependent protein kinase 1 (PDK1) at threonine 308 (Thr308) residue [15], [16]. Phosphorylation of serine 473 (Ser473) residue is a target of the mTOR complex 2 (mTORC2) [17] and DNA-dependent protein kinase (DNA-PK) [18]. Maximal AKT activity is dependent on the phosphorylation status of both Thr308 and Ser473 residues [15].

Upon stimuli or stresses, AKT activity can be modulated via interaction with a number of cytosolic chaperones including heat shock protein 27 (HSP27), HSP70 and HSP90 [19]–[21]. While under ER stress, cell-specific ablation or chronic elevation of GRP78 reduces and elevates AKT phosphorylation respectively [22], [23]. ER stress also attenuates AKT protein translation [24]. In this study, we further demonstrate that ER stress modulates AKT downstream substrates specificity in a severity-dependent manner. ER stress induces GRP78 expression and promotes an interaction between GRP78 and AKT, as shown by an *in situ* proximity ligation assay (PLA) and co-immunoprecipitation, which in turn suppresses Ser473 phosphorylation and thereby modulates substrate specificity. Our results provide a mechanistic explanation on how ER stress may differentially regulate a variety of cellular responses via the AKT pathway in a severity-dependent manner.

## Results

### ER stress modulates AKT downstream substrate specificity in a severity-dependent manner, by suppression of AKT phosphorylation, but not PDK1

**Figure 1:**
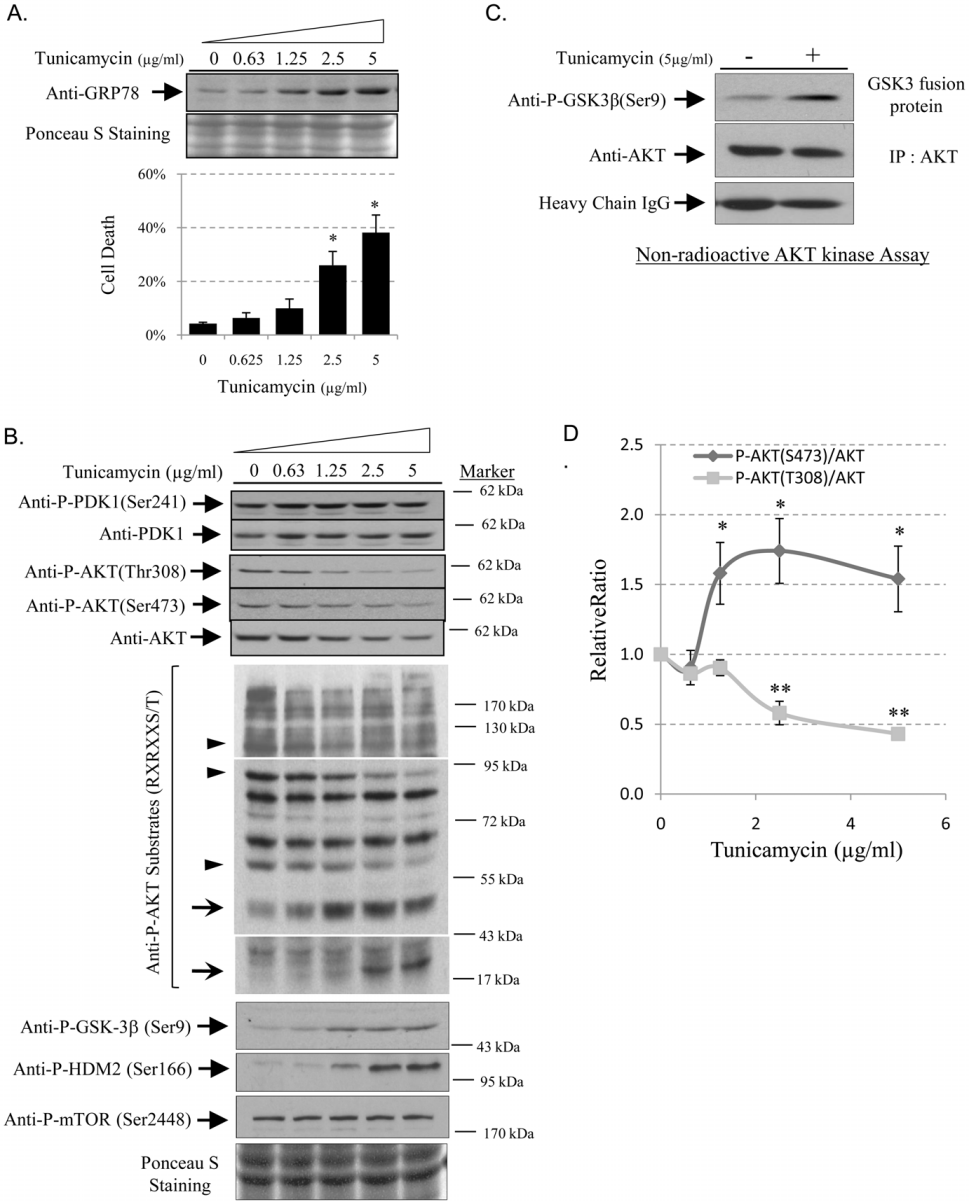
ER stress reduces AKT phosphorylation at Thr308, but increases it at Ser473, and alters target substrate specificity. In a dose-response study of tunicamycin, JEG-3 cells were treated with increasing concentrations of tunicamycin (0, 0.625, 1.25, 25 and 5 µg/ml) for 24 hours. Proteins were isolated for Western blotting analysis for GRP78, AKT, P-AKT(Thr308), P-AKT(Ser473), P-PDK1(Ser241), P-AKT substrate (RXRXXS/T), P-mTOR(Ser2448), P-HDM2(Ser166), and P-GSK-3β(Ser9). Ponceau S staining was used to show equivalent input of cell lysate. Densitometry of band intensity is expressed relative to untreated control (100%). Phosphorylation status is presented as the ratio between phosphorylated and total protein. Data are mean±SEM from 3 to 5 independent experiments. ** and * indicate *P<*0.01 and P<0.05. **A**) Increasing severity of ER stress gradually induces GRP78 expression and cell death. **B**) ER stress suppresses AKT phosphorylation and modulates downstream substrates specificity without affecting PDK1 phosphorylation. Arrowheads and arrows indicate AKT substrate phosphorylation levels going down and going up respectively with increasing ER stress. Because of the different abundances of the AKT substrates, the blots of phospho-AKT substrates necessitated different exposure times. Here, three exposures are merged into a single image in order to view all potential bands. The blot shown is a typical result from 3 independent experiments. **C**) An *in vitro* non-radioactive AKT kinase assay using GSK-3β fusion protein as the substrate showed increased overall AKT activity in tunicamycin-treated cells. A similar result was obtained from a repeat experiment. **D**) Normalisation between phosphorylated AKT and AKT indicates an increase of Ser473 phosphorylation but a decrease at Thr308.

To investigate whether the severity of ER stress affects AKT downstream substrate specificity, we treated human choriocarcinoma, JEG-3 cells with different concentrations of ER stress inducer, tunicamycin. Increased concentration of tunicamycin gradually elevated the severity of ER stress indicated by continuous up-regulation of GRP78 protein and cell death in a dose-dependent manner (Fig. 1A). The treatment also induced a dose-dependent reduction of AKT phosphorylation at both Ser473 and Thr308 residues, but not on PDK1 at Ser241 (Fig. 1B). However, there was an associated reduction in total AKT protein due to attenuation of translation as previously described [24]. To gain an overview of how the severity of ER stress influences AKT downstream target substrate recognition, we used an anti-phospho-AKT substrate (RXRXXS/T) antibody that detects the phosphorylation status of multiple potential AKT substrates. In Figure 1B, at least 5 bands with molecular weights of approximately 110, 95, 60, 45 & 25 kDa were differentially phosphorylated in response to increasing concentrations of tunicamycin (arrows indicate increases; arrowheads indicate decreases). In order to verify the above data, several well-known AKT downstream targets, including phospho-mTOR (Ser2448) [25], phospho-HDM2 (Ser166) [26], and phospho-GSK3β (Ser9) [27] were tested. Based on its molecular weight, we speculated that the protein around 45 kDa could be GSK-3β and an anti-P-GSK-3β specific antibody confirmed an increase in phosphorylation of GSK-3β. There was also an increase of phosphorylation of HDM2 at Ser166 and phosphorylation of mTOR at Ser2448 remained constant (Fig. 1B).

### ER stress differentially regulates AKT phosphorylation at Thr308 and Ser 473

The full activity of AKT depends on the phosphorylation level at both the Thr308 and Ser473 residues as well as on its total protein concentration [15]. Therefore, it was difficult to reveal the activity of AKT when both phosphorylated and total protein forms were reduced simultaneously. Consequently, we measured AKT kinase activity directly using a non-radioactive kinase assay in which a GSK-3 fusion protein is used as the substrate. Equal amounts of AKT proteins were pulled down by AKT antibody from control and treated samples. Surprisingly, we detected an increase in AKT activity despite the reduction in phosphorylation at both Thr308 and Ser473 (Fig. 1C), suggesting a possible increase of relative phosphorylation levels of AKT. Therefore, a normalisation between the phosphorylated and total proteins was performed. As shown in Figure 1D, there was a gradual reduction of Thr308 phosphorylation, reaching approximately 60% in the presence of 5 µg/ml tunicamycin. In contrast, there was an approximately 1.6 fold increase of phosphorylation at Ser473 at 1.25 µg/ml, which remained constant up to 5 µg/ml. Crucially, when the ratio of Ser473/Thr308 was plotted against the dose of tunicamycin on a Log scale, we observed a strong positive correlation (R^2^ = 0.9804) (Fig. S1). Similarly strong positive and negative correlations were observed with the phosphorylation profile of several of the potential AKT substrates identified using the anti-phospho-AKT substrate (RXRXXS/T) antibody (Fig. S1). These data suggest that the severity of ER stress differentially regulates Thr308 and Ser473 phosphorylation, and that the ratio between the two residues could be important in determining AKT's downstream substrate specificity. The next question was how ER stress regulates AKT phosphorylation.

### Down-regulation of GRP78 increases AKT phosphorylation at Ser473, but not Thr308

**Figure 2:**
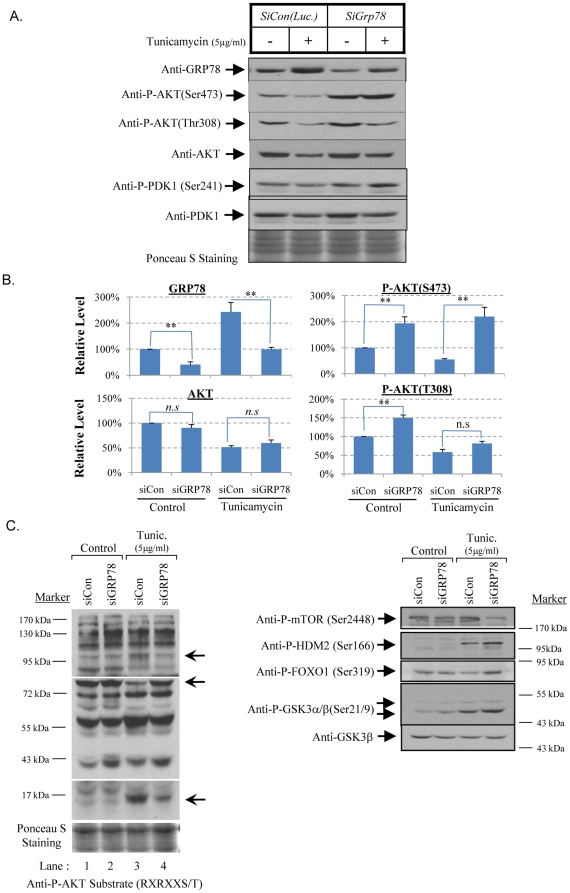
Knock-down of ER stress-induced GRP78 expression by *siGRP78* restored AKT phosphorylation at Ser473, but not at Thr308, and altered AKT substrates specificity. Cells were transfected with either *siCon* or *siGRP78* RNA duplexes for 24 hour before treatment with tunicamycin for 24 hour. Proteins were extracted for immunoblot analysis with GRP78, P-PDK1(Ser241), PDK1, P-AKT(Thr308), P-AKT(Ser473), AKT, P-AKT substrate (RXRXXS/T), P-mTOR(Ser2448), P-HDM2(Ser166), P-FOXO1(Ser319) and P-GSK-3α/β(Ser21/9). Densitometry of band intensity is expressed relative to *siCon* untreated control (100%). Phosphorylation status is presented as the ratio between phosphorylated and total protein. Data are mean±SEM for 3 independent experiments. ** indicates *P≤*0.01; *n.s* indicates non-significant change.** A & B**) Down-regulation of GRP78 elevates Ser473 phosphorylation but not at Thr308.** C**) Knock-down of GRP78 alters AKT downstream substrates recognition. Arrows indicate the substrates changed their phosphorylation pattern in tunicamycin-treated siGRP78 cells.

The ER-specific chaperone, GRP78, has recently been reported to regulate AKT phosphorylation at the Ser473 residue [22], [23]. Additionally, it is also known that ER stress induces expression of GRP78 in the severity-dependent manner [12], [28], and so this appears a good candidate for further investigation. Therefore, a small interference RNA (siRNA) was used to knock-down GRP78 induced by ER stress in order to examine the effects on the phosphorylation status of AKT. JEG-3 cells were transfected with either *siLuciferase (siCon)* for control or two different sets of *GRP78* siRNA duplexes which were used to eliminate off-target effects. Compared to *siCon*, *siGRP78* RNA duplex reduced GRP78 by more than 50% in control and tunicamycin treated cells (Figs. 2A & B; Fig. S2). No increase in cell death was detected in GRP78 knock-down cells after 72 hour, but there was an increase of expression of GRP94 and phosphorylation of eIF2α, suggesting induced ER stress but at a sublethal level (data not shown). Knock-down of GRP78 significantly elevated phosphorylation levels of AKT at both Ser473 and Thr308 by ∼2 and ∼1.5 fold respectively without affecting total AKT protein level (Fig. 2B). Upon tunicamycin treatment, reduction of GRP78 greatly increased AKT phosphorylation at Ser473 by ∼3.6 fold (Figs. 2A & B). In contrast, phosphorylation at the Thr308 residue was not significantly changed. Knock-down of GRP78 did not affect the total AKT protein concentration, or the phosphorylation of PDK1 at Ser241 (Figs. 2A & B).

### Increased Ser473 phosphorylation modulates AKT substrate specificity

To investigate whether elevated Ser473 phosphorylation upon *siGRP78* treatment alters AKT downstream target recognition, the anti-phospho-AKT substrate (RXRXXS/T) antibody was again used. At least 3 bands with differential intensity were detected (Fig. 2C, indicated by arrows). Again, phospho-specific antibodies for known AKT substrates including P-mTOR(Ser2448), P-HDM2(Ser166), P-FOXO1(Ser319) [29]; and P-GSK-3β(Ser9) were used to verify the finding. In the *siGRP78* transfected cells, tunicamycin treatment reduced P-mTOR (Ser2448) while it increased P-HDM2 (Ser166), P-FOXO1 (Ser319) and P-GSK3α/β (Ser21/9) phosphorylation levels compared to *siCon* (Fig. 2C, left panel). Thus, ER stress regulates AKT Ser473 phosphorylation possible via GRP78 that in turn modulates the AKT target substrate specificity. We therefore next addressed how GRP78 is able to regulate AKT Ser473 phosphorylation.

### ER stress facilitates an interaction between GRP78 and AKT *in vivo*

**Figure 3:**
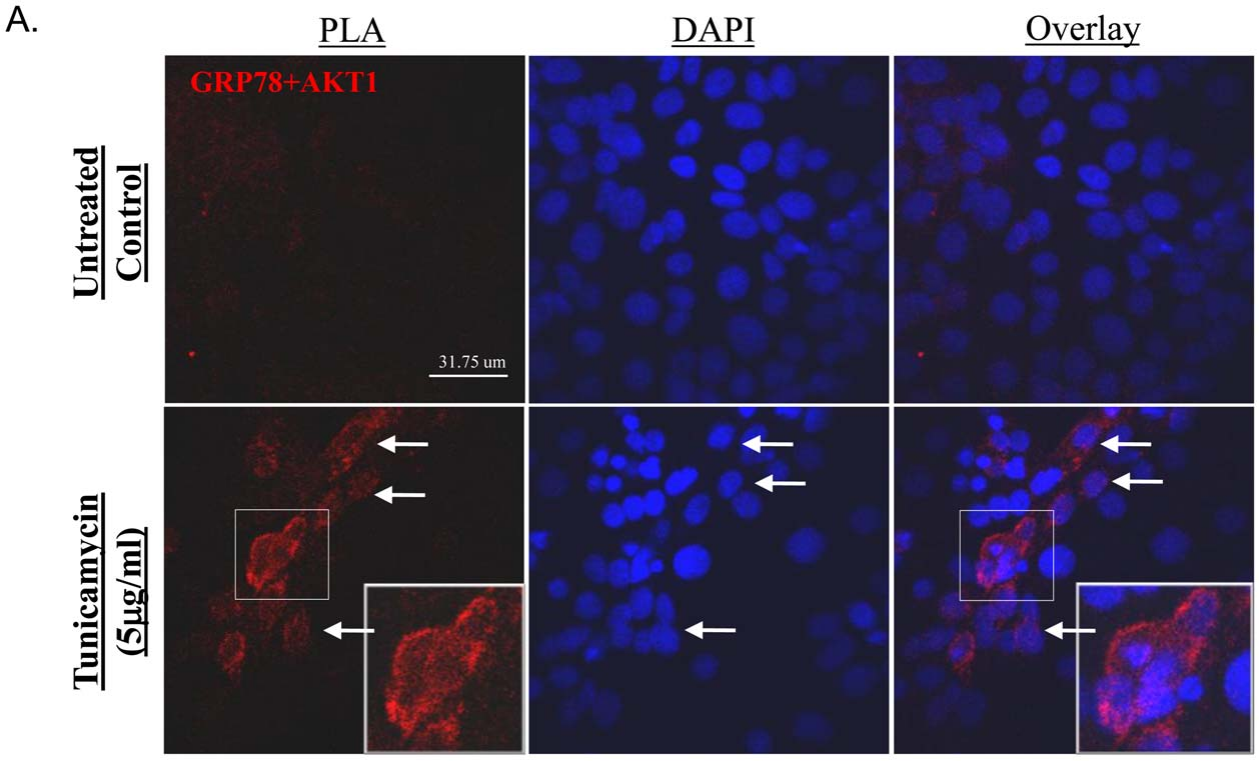
The interaction between AKT and GRP78 occurs *in vivo*. After tunicamycin treatment, JEG-3 cells were fixed in 100% methanol and subjected to a DuoLink Proximity Ligation Assay *in situ* and confocal microscopy. Arrows indicate cells staining positive with normal nuclear morphology, whereas stronger staining was seen in cells with condensed nuclei. All images were a single optical section taken with a 60X objective using the same PMT, gain, and offset setting. Scale bar = 31.75 µm for all panels.

To test whether there is any interaction between GRP78 and AKT, an *in situ* Proximity Ligation Assay (PLA), a technique that detects an interaction between two proteins *in vivo* [30], was employed. As shown in Figure 3A, positive staining was observed in tunicamycin-treated cells and the majority of staining was at the plasma membrane of cells. The staining was weakest in cells with normal nuclear morphology, and strongest in those with condensed nuclei (Fig. 3A). To eliminate false positives, an anti-HA-tag antibody that does not recognize any mammalian proteins was used in conjunction with anti-AKT1, and only a weak background signal was detected (Fig. S3). These data suggest that the majority of the association between GRP78 and AKT occurs in the plasma membrane, consistent with the findings of Zhang *et al.* of relocation of GRP78 to the plasma membrane upon ER stress [4].

### The interaction of GRP78 with AKT prevents Ser473 phosphorylation

**Figure 4:**
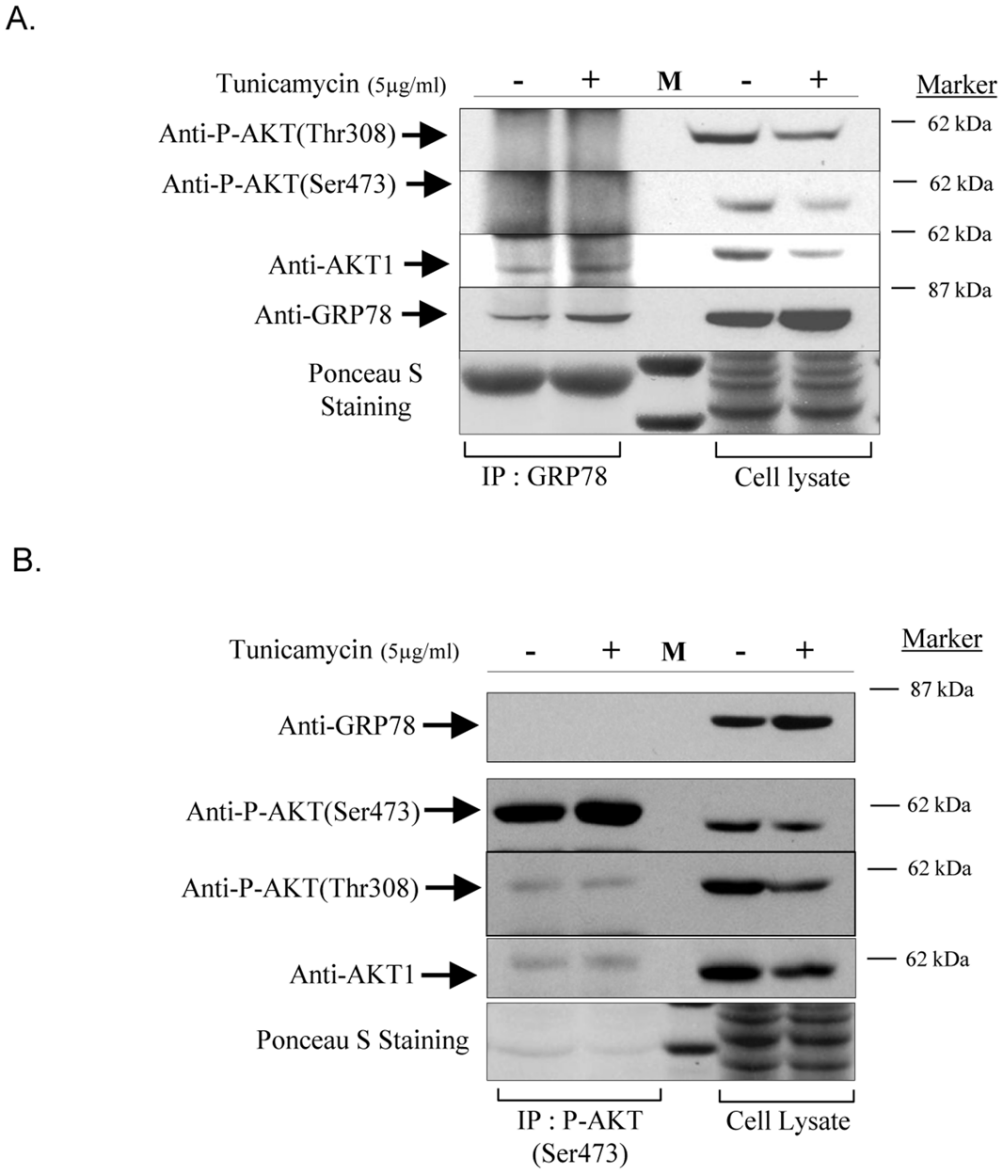
Association of GRP78 with AKT prevents AKT phosphorylation at Ser473 but not at Thr308. **A**) Loss of AKT phosphorylation at Ser473 and Thr308 in the GRP78-IP complex. AKT was pulled down by anti-GRP78 (N-20) antibody and immunoblotted for P-AKT(Ser473), P-AKT (Thr308), AKT1 and GRP78 antibodies. **B**) No GRP78 was pulled down by P-AKT(Ser473) antibody.

To elucidate whether the binding of GRP78 to AKT blocks the phosphorylation of Ser473, co-immunoprecipitation of GRP78 followed by immunoblotting with P-AKT(Ser473) and vice versa was performed. As shown in Figure 4A, no AKT phosphorylated at either Ser473 or Thr308 was detectable in the GRP78-immunoprecipitated (GRP78-IP) complex, while immunoblotting for AKT1 revealed a strong signal in the GRP78-IP product of both control and tunicamycin treated samples. As the band intensity of AKT1 was similar in the lanes containing the input cell lysate and GRP78-IP product in the tunicamycin treated samples, it is very unlikely that phosphorylated AKT was undetectable due to less protein input. Interestingly, the AKT in GRP78-IP product had a slightly lower molecular weight than in cell lysate. Change in mobility (a “band shift”) is a common feature of phospho-proteins or kinases upon phosphorylation (Fig. S4).

To further support the above observation, reciprocal immunoprecipitation using anti-phospho-AKT(Ser473) and immunoblotting with anti-GRP78 was performed. As shown in Figure 4B there was no GRP78 signal following P-AKT(Ser473) immunopreciptitation, while P-AKT(Ser473) immunoblotting revealed a very strong signal. Immunoblotting for P-AKT(Thr308) also revealed a signal in P-AKT(Ser473)-IP products, but to a much lesser extent. These data strong suggested that GRP78 binds to non-phosphorylated AKT.

### Binding of GRP78 to AKT is likely a specific phenomenon in a variety of cells in response to ER stress

**Figure 5:**
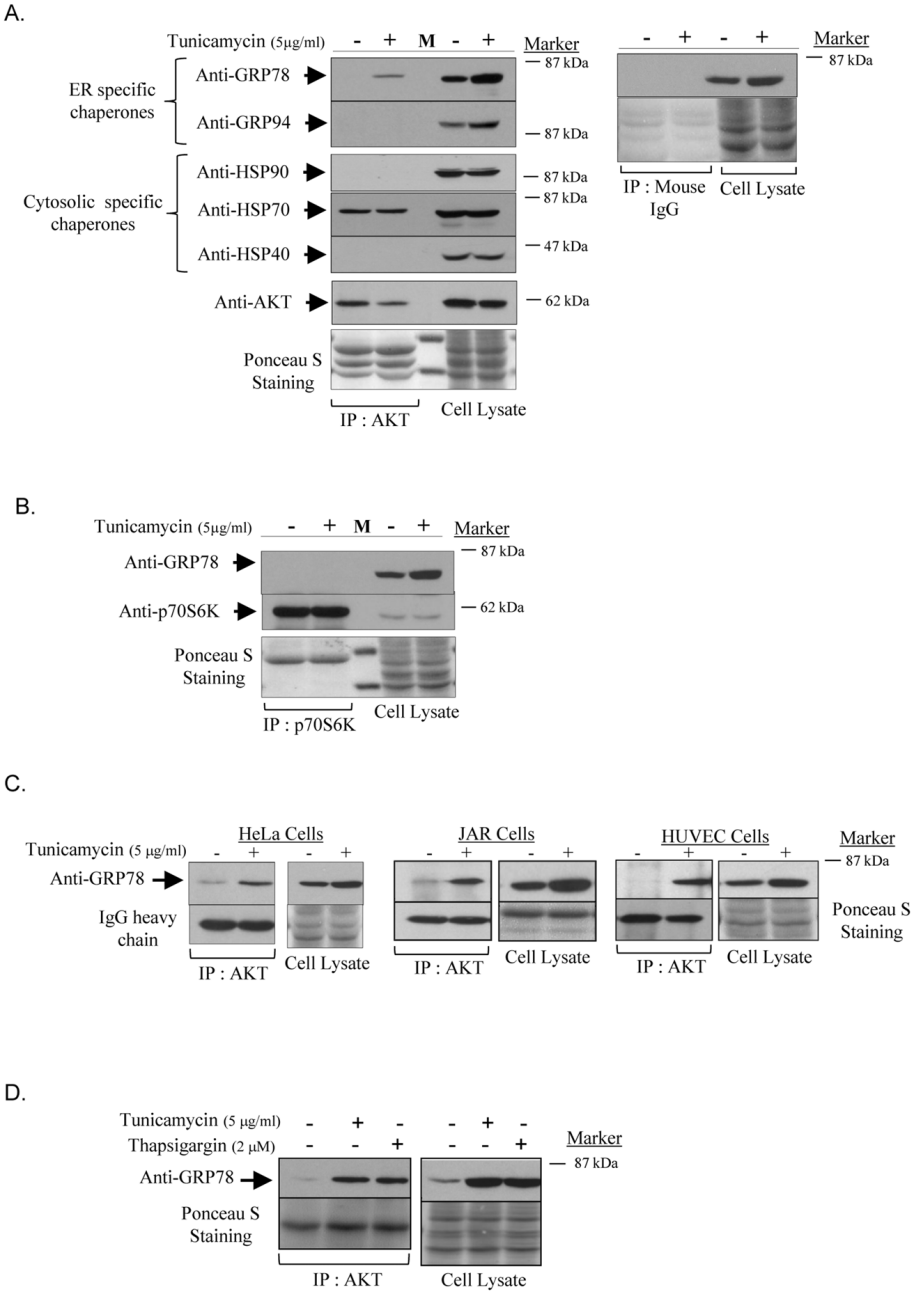
The interaction between AKT and GRP78 is likely a specific phenomenon upon ER stress in a variety of cell types. JEG-3 cells were treated with tunicamycin for 24 hour before protein isolation for immunoprecipitation followed by immunoblotting. Ponceau S staining and IgG heavy chain were used to show both equivalent input of cell lysates and antibodies respectively. **A**) AKT does not interact with many chaperones. AKT immunoprecipitated products were immunoblotting with antibodies against GRP94, HSP90, HSP70 and HSP40. Mouse IgG was used as a negative control and no interaction was observed. **B**) GRP78 does not bind to other AGC family member, p70 S6 kinase. P70 S6 kinase immunoprecipitated products were immunoblotting with GRP78 and p70 S6 kinase. **C**) The interaction between GRP78 and AKT is also found in HeLa, JAR and primary HUVECs. **D**) Binding of GRP78 with AKT is observed following another ER stress inducer, thapsigargin.

AKT interacts with several HSPs under stresses/stimuli [19]–[21]. Therefore, we also examined whether AKT binds to other molecular chaperones including another ER-specific chaperone, GRP94, the cytosolic chaperones heat shock proteins 70 & 90 (HSP70 & HSP90), and co-chaperone HSP40/DnaJ. Treatment with tunicamycin greatly elevated GRP94, but not HSP90, HSP70, or HSP40. Crucially, up-regulation of GRP94 did not promote any interaction with AKT (Fig. 5A), and no interactions with HSP90 and HSP40 were observed. Although an interaction between HSP70 and AKT was detected, the degree was similar in both control and tunicamycin treated AKT-IP products (Fig. 5A).

Next, we tested the specificity of GRP78 binding to AKT. The p70 S6 kinase (S6K1) was selected because it is also a member of the AGC kinase family and shares similar protein structure to AKT, consisting of two highly conserved Ser/Thr residues and the hydrophobic motif in the catalytic domain at the C-terminus [31]. S6K1 immunoprecipitation pulled down a large amount of S6K1, but no association of GRP78 was observed (Fig. 5B).

Furthermore, we examined whether the binding of GRP78 to AKT is specific to JEG-3 cells under tunicamycin treatment, or if it is a general phenomenon in a variety of cells. The interaction was observed in HeLa, another human choriocarcinoma cell, (JAR), and primary human umbilical vascular endothelial cells (HUVECs), excluding cell type specific effect (Fig. 5C). To eliminate a drug-specific effect, thapsigargin, another ER stress inducer was used, and was found to enhance the interaction (Fig. 5D). These results confirm that ER stress promotes the interaction between GRP78 and AKT in a variety of cell types.

## Discussion

ER stress or the UPR contributes to the pathophysiology of many human disorders that demonstrate contrasting outcomes, such as the promotion of cell survival in cancer [32], slowing down of cell proliferation in intrauterine growth restriction [12], and facilitation of apoptosis in neurodegenerative diseases [33]. How ER stress mediates such contrasting cellular behaviours is largely unknown. Recent publications from our own and other laboratories suggest that it may be via AKT signalling [22]–[24]. AKT signalling regulates a wide range of cellular processes through phosphorylation of a variety of downstream target substrates, including mTOR, FOXO1, HDM2 or MDM2, BAD, p21Cip, GSK3 and eNOS etc. AKT therefore represents a suitable pivotal kinase for the ER stress response to target. The mechanism for AKT to recognise its downstream substrates is reliant on the phosphorylation status of the Ser473 residue [34], [35]. Here, our results not only demonstrated ER stress induced phosphorylation of Ser473, it altered AKT substrate recognition profile in a severity-dependent manner. Additionally, we propose a new rationale that AKT substrates specificity is likely dependent on the ratio between the phosphorylation status of Ser473 and Thr308, rather than Ser473 alone. This speculation is supported by our data showing strong correlations between the severity of ER stress and the ratio of Ser473/Thr308, and the phosphorylation profile of several AKT substrates in a severity-dependent manner (Fig. S1), but not with phosphorylation level of Ser473 alone (Fig. 1D). The rationale is further supported by the GRP78 knock-down study, in which down-regulation of GRP78 increased the ratio of Ser473/Thr308 by elevating Ser473 phosphorylation without affecting Thr308. This change again altered AKT substrate specificity.

A common feature of molecular chaperones is to bind to client proteins in order to serve as buffering agents by masking the functional domain or altering their conformation [36]. Binding of HSP27 to AKT facilitates its phosphorylation by promoting binding of activating kinase [19]. HSP90 forms a complex with AKT, thereby preventing dephosphorylation [20], [37], while HSP70 regulates AKT protein degradation [21]. These findings strongly suggest that apart from activating kinases and phosphatases, AKT activity can be modulated via the interaction with molecular chaperones. Here, our study revealed that upon ER stress, GRP78 binds to AKT and modulates AKT substrate specificity through regulation of Ser473 phosphorylation. The *in situ* PLA showed that AKT comes into close approximation *in vivo*, suggesting a physical interaction. However, although the technique detects target proteins within 40 nm of each other, we cannot be certain whether this is a direct binding or if it requires other factors. The binding of GRP78 to AKT prevents Ser473 phosphorylation and can be reversed by knock-down of GRP78. In addition, ER stress appeared to have different effects on Ser473 phosphorylation in different cell types. We observed an increase of Ser473 phosphorylation in the JAR cells upon tunicamycin treatment, but suppressed in the primary HUVECs (Fig. S5). Nevertheless, knock-down of GRP78 in both JAR and HUVECs also elevated Ser473 phosphorylation, eliminating JEG-3 cell-specific effects.

GRP78 recognises and binds to the hydrophobic motifs of client proteins [38]. AKT contains a single hydrophobic motif (from residues 469–474 in AKT1), where the Ser473 residue is located, near the C-terminus [39], which may provide a binding site for GRP78. The binding of GRP78 to AKT, therefore, could affect the accessibility of Ser473 for the activating kinases. This rationale was supported by the results presented in Figures 2 and 4. An interaction between GRP78 and AKT has also been demonstrated in a proteomic approach when searching for substrates of AKT phosphorylation in mesangial cells [40]. Constructs with deleting mutants of both GRP78 and AKT will be required to identify the amino acid sequences involved in the binding in the future studies. The molecular weight of GRP78 pulled down by AKT is around 78 kDa, eliminating possible interaction with GRP78va which molecular weight is about 62 KDa.

The question arises as to how an ER resident chaperone is able to interact with a cytosolic kinase. The PLA images suggested that the GRP78-AKT complexes were close to the plasma membrane, consistent with the finding of Zhang *et al.* that a proportion of GRP78 relocates to the plasma membrane in response to ER stress [4]. Although GRP78 is generally a hydrophilic protein, it also exhibits some properties of a transmembrane protein as it contains several hydrophobic regions [7]. The staining was weak in cells with normal morphology but became stronger in the cells with condensed nuclei, suggesting that the interaction might facilitate cell death. Interestingly, plots of the amount of GRP78-AKT immune-complex and the percentage of cell death against the increasing severity of ER stress revealed strong positive relationships (Fig. S6).

**Figure 6:**
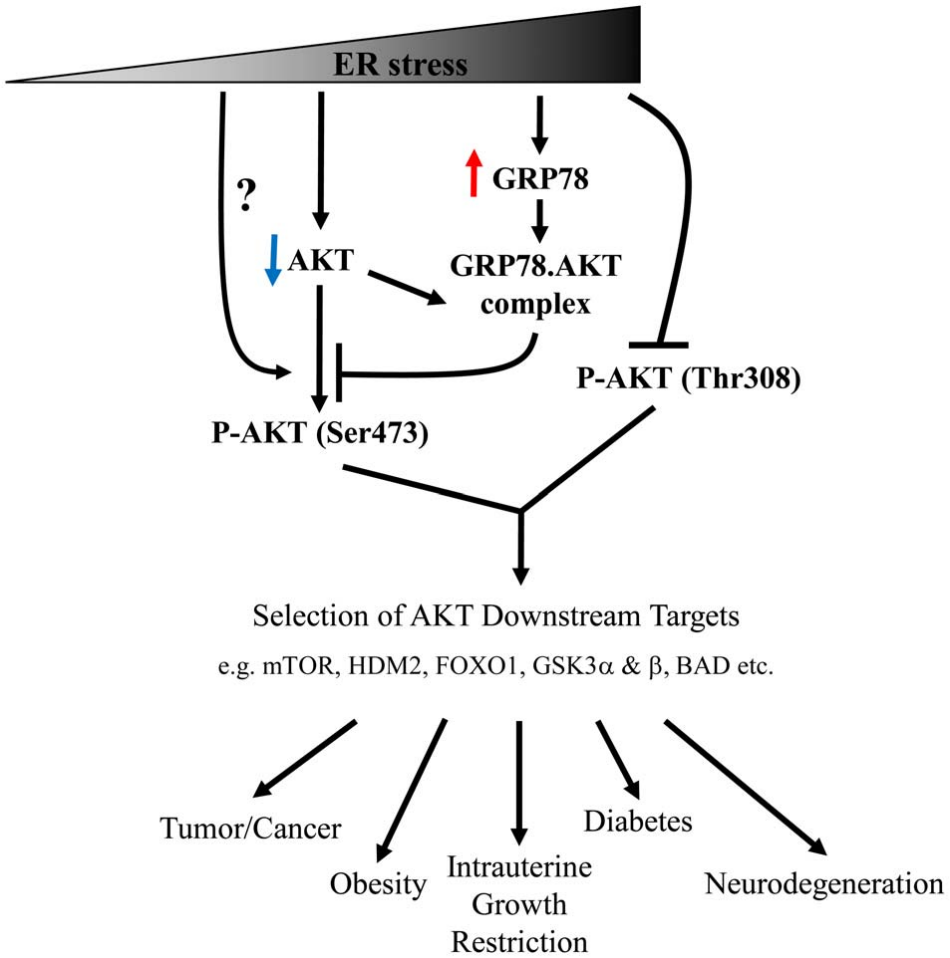
A schematic model proposing how the severity of ER stress might modulate AKT target substrate specificity, and the consequent pathologies.

To conclude, our data demonstrate that ER stress modulates AKT target substrate specificity in a severity-dependent manner. The molecular mechanisms underlying this phenomenon are still far from clear, although an interaction between GRP78 and AKT could provide one explanation. As ER stress alters many signalling pathways, we cannot exclude the possibility that other pathways altered by ER stress also contribute to the change of AKT phosphorylation. Taken together, these findings demonstrate a critical mechanism by which ER stress modulates the AKT signalling pathway in order to differentially control cellular processes. A schematic diagram summarising the above results is presented in Figure 6. With the increasing recognition of an association between ER stress and human diseases, these findings provide new insights for the design of pharmacological interventions aimed at either inducing apoptotic death in cancer cells or conversely promoting cell survival in neurodegenerative diseases.

## Materials and Methods

### Chemicals and antibodies

Tunicamycin, thapsigargin, cycloheximide, poly-L-lysine, 2% gelatin solution, saponin, bovine serum albumin and anti-β-actin antibody were from Sigma-Aldrich (Poole, UK). BAF [boc-aspartyl(OMe)-fluoromethylketone] was from Cambridge Bioscience (Cambridge, UK). Anti-phospho-AKT (Ser473), anti-AKT, anti-AKT1, anti-phospho-mTOR (Ser2448), anti-phospho-HDM2 (Ser166), anti-phospho-FOXO1 (Ser319), anti-phospho-GSK3α/β (Ser21/9), immobilized AKT (1G1) antibodies (bead conjugated), immobilized phospho-AKT (Ser473) (D9E) antibodies (bead conjugated) and immobilized IgG mouse (bead conjugated) were from Cell Signaling Technology (New England Biolabs Ltd, UK). Anti-p70 S6 kinase, Anti-phospho-AKT (Thr308), Anti-AKT1, and anti-GRP78 (N-20) were from Santa Cruz Biotechnologies (Insight Biotechnology Ltd, UK). Anti-HSP70 and Anti-HSP90 were from ENZO Life Science (Exeter, UK). Anti-GRP94 and Anti-HSP40 antibodies were from Abcam (Cambridge, UK). Anti-GRP78 antibodies were from Abcam (Cambridge, UK) for immunocytochemistry or from BD Transduction Laboratories (Oxford, UK) for Western blotting.

### Cell culture

Human choriocarcinoma JEG-3 cells were a gift from Professor Ashley Moffett (University of Cambridge, UK). Cells were grown as in previous described [24]. For experiments, after passage, cells were grown in serum containing medium for 2 days until they reached full confluency. Cells were then rinsed once with serum-free medium before incubation with serum-free medium containing any drugs for the desired time spans at 37°C in a 5% CO_2_ atmosphere.

### Co-immunoprecipitation

Cell lysate preparation and protein concentration determination were carried out as previously described [24]. Both AKT and P-AKT(Ser473) immunoprecipitations were performed following the manufacturer's protocol (Cell Signaling Technologies). Briefly, 500 µg of protein from the whole cell lysate were diluted with lysis buffer to 200 µl, before addition of 20 µl of immobilized antibodies bead slurry and incubation overnight at 4°C with gentle rocking. After extensive washing with lysis buffer to remove residual proteins, the beads were mixed with protein gel loading buffer and boiled for 10 minutes, before resolving by SDS-PAGE and immunoblotting with an anti-GRP78 specific antibody.

GRP78 or p70S6 kinase immunoprecipitations were performed as follows. Briefly, 500 µg of whole cell lysate was adjusted to 200 µl with lysis buffer. To pre-clear the cell lysate, 10 µl of protein A/G agarose bead slurry (Santa Cruz Biotechnologies) was added and incubated for an hour at 4°C with gentle rocking. After a brief spin, the supernatant was transferred to a new eppendorf, and 6 µg of anti-GRP78 (N-20) antibody was added and incubated overnight at 4°C. 30 µl of protein A/G agarose bead slurry was added to the mixture and incubated for a further 4 hours at 4°C with gentle rocking. After extensive washing with lysis buffer, proteins were released by boiling the beads in gel loading buffer and resolved by SDS-PAGE. This was followed by immunoblotting with anti-AKT1, anti-phospho-AKT (Ser473), anti-phospho-AKT (T308), and anti-GRP78.

### Western Blot

Protein expression and kinase phosphorylation levels were measured by Western blotting were carried out as previously described [24]. Equivalent amounts of protein were resolved by SDS-PAGE, blotted onto nitrocellulose (0.2 µm) and analyzed by enhanced chemiluminescence (ECL) (Amersham Bio-sciences, UK) using Kodak X-OMAT film (Sigma-Aldrich). Films were scanned using a flat-bed scanner (Cannon 8000F) and intensities of the bands representing phospho- and total kinase forms were determined from two or three different exposures (within the linear detection range) using Image J analysis software (Freeware).

### Small RNA Interference

The siRNA duplexes used in the study were from Dharmacon (Thermo Scientific, UK). For GRP78, either the siGENOME SMARTpool GRP78 (M-008198-02), or 4 individual siRNA duplexes from the siGENOME GRP78 were used, including seq. 1 CCACCAAGAUGCUGACAUU (D-008198-03); seq. 2 GAAAGGAUGGUUAAUGAUG (D-008198-04); seq. 3 CGACUCGAAUUCCAAAGAU (D-008198-05); seq. 4 CAGAUGAAGCUGUAGCGUA (D-008198-18). For non-targeting siRNA controls, siRNA duplexes targeting firefly luciferase mRNA, and the siGENOME non-targeting siRNA #4 (D-001210-04-05) (Dharmacon) were used. SiPortAmine transfection reagent was purchased from Applied Biosystems (Warrington, UK). Transfection of siRNA was carried out according to the manufacturer's instructions. The day before transfection, cells were seeded at a density that would reach ∼70% confluency the next day. Briefly, 10 µl of SiPortAmine transfection reagent was diluted with 100 µl of OPTIMEM (Invitrogen Ltd, Paisley, UK) and incubated at room temperature for 10 minutes. 15 µl of 10 µM siRNA was diluted with 100 µl of OPTIMEM, and the two mixtures were mixed and incubated at room temperature for 10 minutes before being applied to the cells. After 24 hour of incubation, the efficiency of the different GRP78 siRNA sequences was determined by Western blot analysis using anti-GRP78 specific antibody (Fig. S2). Based on the results, either duplex seq. 3 or seq. 4 was used for the subsequent studies.

### Proximity Ligation Assay *in situ*

JEG-3 cells were grown on poly-L-lysine and 1% gelatin coated coverslips until confluent in serum-free RPMI 1640 medium, before treatment with 5 µg/ml tunicamycin for 24 hour. Cells were fixed with 100% methanol at −20°C for 20 minutes, permeabilized with 0.1% saponin in PBS containing 1% bovine serum albumin (Sigma-Aldrich) for 20 minutes followed by incubation with anti-GRP78 (anti-rabbit) and anti-AKT1 (anti-mouse) for overnight at 4°C followed by 1 hour at room temperature. To detect primary antibodies with the *in situ* proximity ligation assay (PLA), the PLA probes mouse PLUS and rabbit MINUS (Abnova, UK) were added at a 1∶5 dilution in antibody dilution buffer (Olink Bioscience, Sweden) for 60 min at 37°C. After washing the coverslips with PBST three times, the probe was detected using *in situ* PLA detection kit 613 (Olink Bioscience) according to the manufacturer's instructions. The coverslip was left to dry before mounting with VECTASHEILD anti-fade medium containing DAPI (Vector Laboratories Ltd, UK). Images were captured using a Leica confocal microscope (Leica TCS-NT,). All images presented were from a single optical section, taken under 63X objective with using the same PMT, gain and offset settings.

### Cell Viability Assay

Cell viability assay was performed using a costaining with two nuclear dyes: Hoechst 33342 (Sigma-Aldrich) and propidium iodide (PI) (Sigma-Aldrich) as previous described [24].

### AKT kinase assay

The non-radioactive AKT kinase assay (Cell Signaling Technology) was performed as previously described [24].

### Statistical Analysis

All experiments were repeated independently twice or more, the data used for statistical analysis were repeated independently at least 3 times or more. Differences between means were tested using a two-tailed Student's *t* test, with *P-*value of lower than 0.05 being considered significant.

## Supporting Information

Figure S1 **A positive or negative correlation exists between the ratio of P-AKT(Ser473/Thr308) and AKT substrate phosphorylation profiles in response to increasing severity of ER stress.** Densitometry of band intensity is expressed relative to untreated control (100%). The graph presents a Log scale.
(TIF)

Figure S2 **Potency of different small interference RNA duplexes specific for** ***GRP78*** **mRNA in the suppression of ER stress-induced GRP78 protein expression.** Cells were transfected with 4 different siRNA sequences for *GRP78* mRNA, a *siGRP78* pool which contains those 4 sequences in equal proportion or siCon which is a siRNA sequence directed against luciferase, following 24 hr incubation before treatment with tunicamycin for an additional 24 hour. Proteins were extracted and immunoblotted for GRP78. Ponceau S staining was used to indicate equal loading of proteins.
(TIF)

Figure S3 **Negative control for in situ PLA assay.** Cells were fixed and probed with anti-HA-tag and anti-AKT1 antibodies. All images are a single optical section taken with a 60X objective using the same PMT, gain, and offset setting. Scale bar = 31.75 um.
(TIF)

Figure S4 **Mobility band shift of AKT under different phosphorylation status.** Cells were treated with 37.5 µM LY294002 for 24 hour. Protein was harvested and analysed by SDS-PAGE followed by immunoblotting with anti-AKT antibody.
(TIF)

Figure S5 **Suppression of ER stress induced GRP78 by s** ***iGRP78*** **enhances AKT phosphorylation at both Thr308 and Ser473, and downstream signalling in JAR and HUVECs.** Cells were transfected with either *siCon* or *siGRP78* RNA duplexes for 24 hour before tunicamycin treatment for an additional 24 hour. Proteins were resolved in SDS-PAGE and immunoblotted for GRP78, P-AKT(Ser473), AKT, GSK3β and β-actin. **A**) JAR cells; **B**) HUVECs.
(TIF)

Figure S6 **The degree of interaction between GRP78 and AKT is direct proportional to the severity of ER stress.** **A)** A dose-response study of tunicamycin. JEG-3 cells were treated with different concentrations of tunicamycin (0, 0.625, 1.25, 25 and 5 µg/ml) for 24 hour. Proteins were isolated for immunoprecipitation with AKT (1G1) antibody, followed by immunoblotting for GRP78 and AKT. Ponceau S staining was used to show both equivalent input of antibody (IgG heavy chain) and total protein. **B**) A graph plotted between the amount of AKT-GRP78 immuno-complex obtained from (A) and the percentage of cell death against the concentration of tunicamycin on a Log scale.
(TIF)
