# Supplementary figures and images for: Regulation of AKT Phosphorylation at Ser473 and Thr308 by Endoplasmic Reticulum Stress Modulates Substrate Specificity in a Severity Dependent Manner
Source: PLoS One. 2011 Mar 21;6(3):e17894. doi: 10.1371/journal.pone.0017894 (PMC3061875; doi:10.1371/journal.pone.0017894)

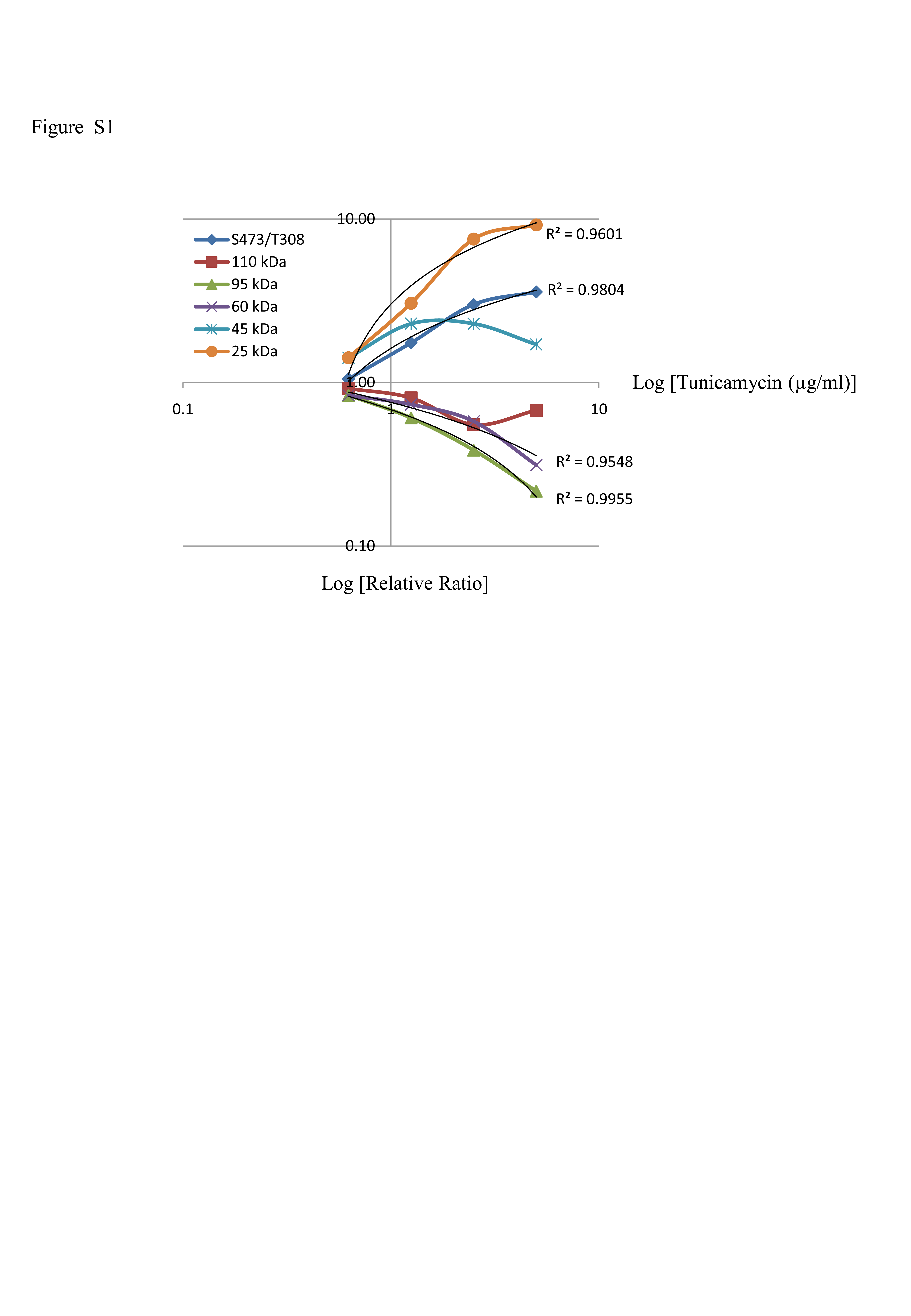

Supplement: Figure S1 — A positive or negative correlation exists between the ratio of P-AKT(Ser473/Thr308) and AKT substrate phosphorylation profiles in response to increasing severity of ER stress. Densitometry of band intensity is expressed relative to untreated control (100%). The graph presents a Log scale. (TIF) [file pone.0017894.s001.tif]

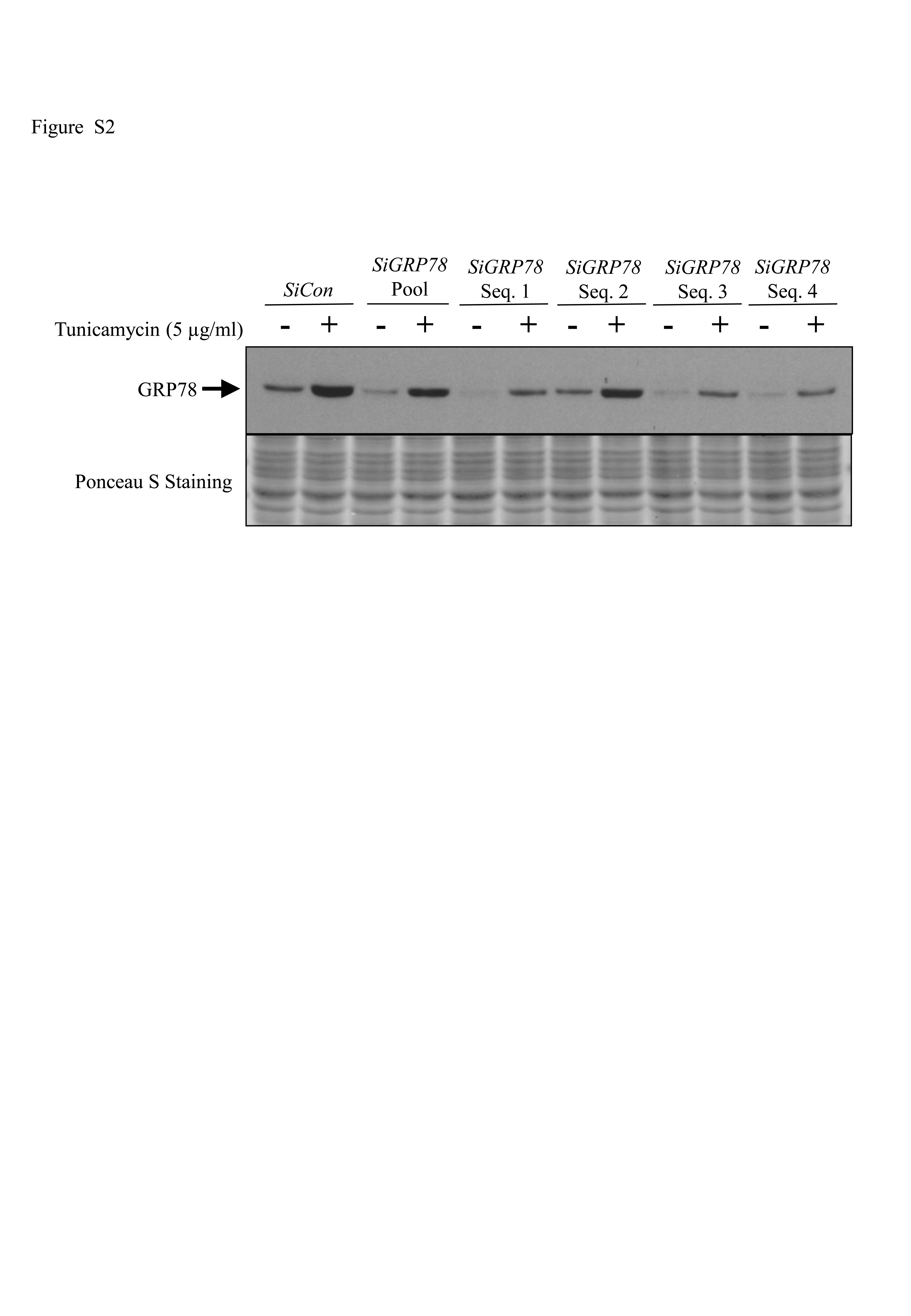

Supplement: Figure S2 — Potency of different small interference RNA duplexes specific for GRP78 mRNA in the suppression of ER stress-induced GRP78 protein expression. Cells were transfected with 4 different siRNA sequences for GRP78 mRNA, a siGRP78 pool which contains those 4 sequences in equal proportion or siCon which is a siRNA sequence directed against luciferase, following 24 hr incubation before treatment with tunicamycin for an additional 24 hour. Proteins were extracted and immunoblotted for GRP78. Ponceau S staining was used to indicate equal loading of proteins. (TIF) [file pone.0017894.s002.tif]

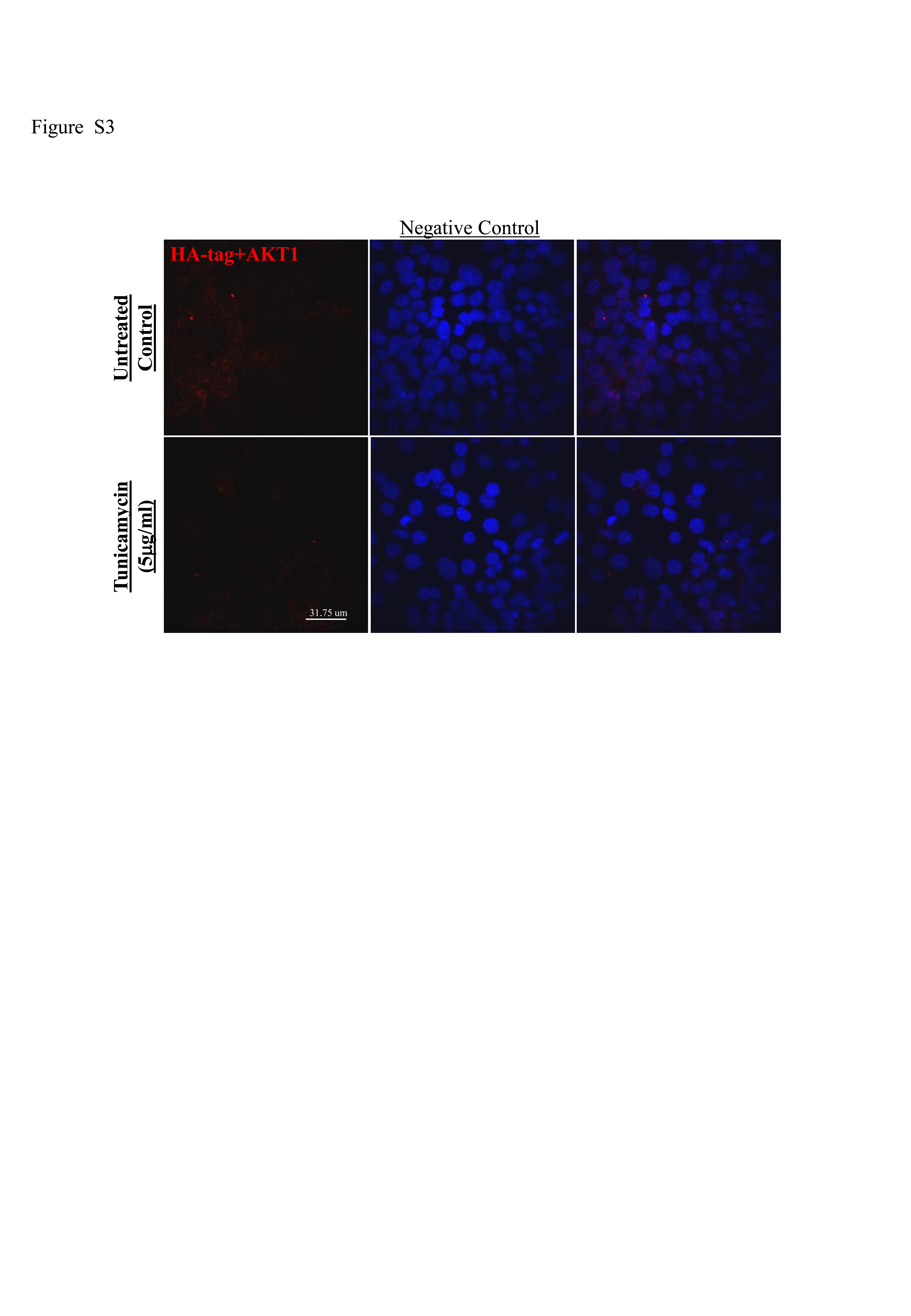

Supplement: Figure S3 — Negative control for in situ PLA assay. Cells were fixed and probed with anti-HA-tag and anti-AKT1 antibodies. All images are a single optical section taken with a 60X objective using the same PMT, gain, and offset setting. Scale bar = 31.75 um. (TIF) [file pone.0017894.s003.tif]

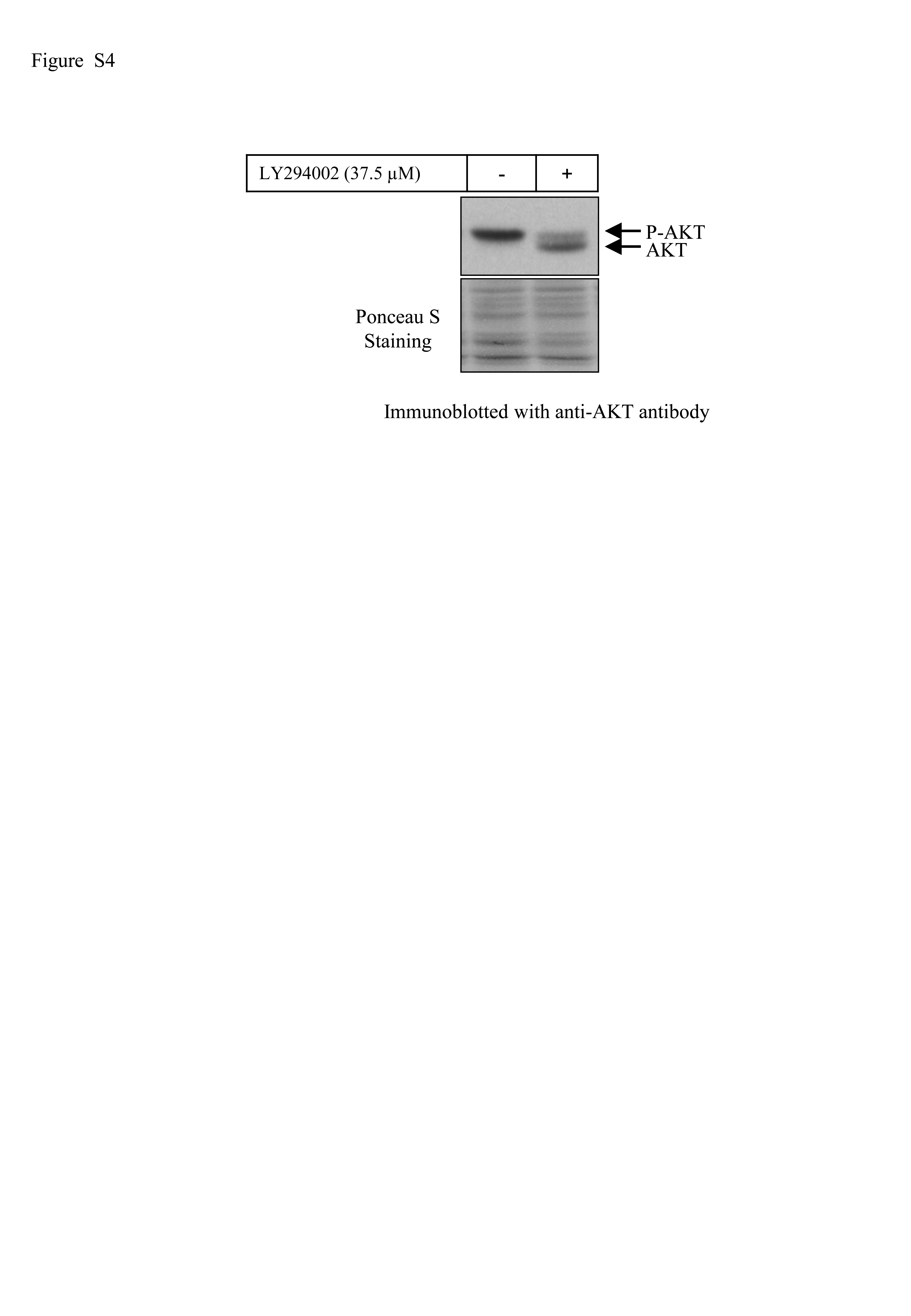

Supplement: Figure S4 — Mobility band shift of AKT under different phosphorylation status. Cells were treated with 37.5 µM LY294002 for 24 hour. Protein was harvested and analysed by SDS-PAGE followed by immunoblotting with anti-AKT antibody. (TIF) [file pone.0017894.s004.tif]

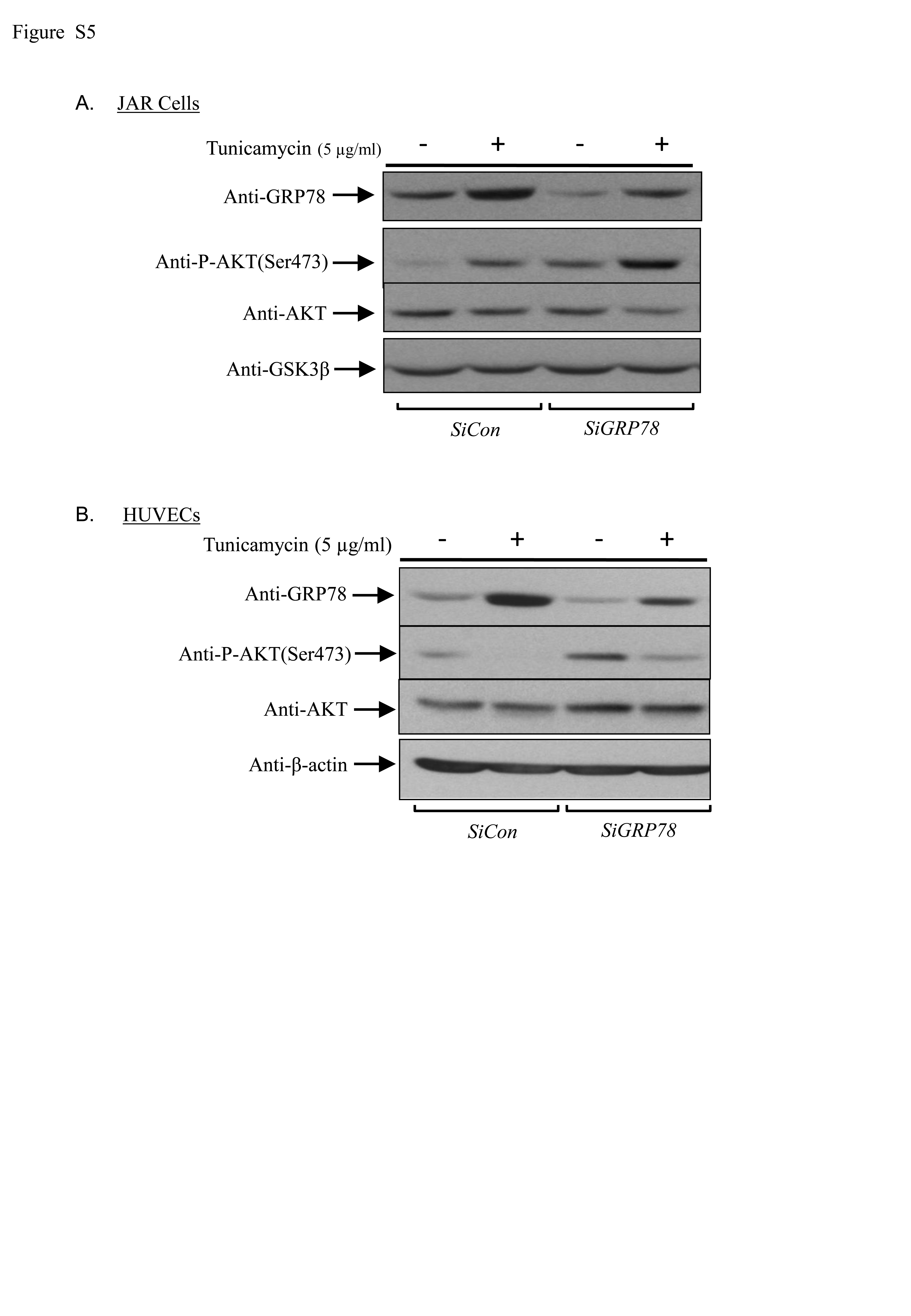

Supplement: Figure S5 — Suppression of ER stress induced GRP78 by s iGRP78 enhances AKT phosphorylation at both Thr308 and Ser473, and downstream signalling in JAR and HUVECs. Cells were transfected with either siCon or siGRP78 RNA duplexes for 24 hour before tunicamycin treatment for an additional 24 hour. Proteins were resolved in SDS-PAGE and immunoblotted for GRP78, P-AKT(Ser473), AKT, GSK3β and β-actin. A) JAR cells; B) HUVECs. (TIF) [file pone.0017894.s005.tif]

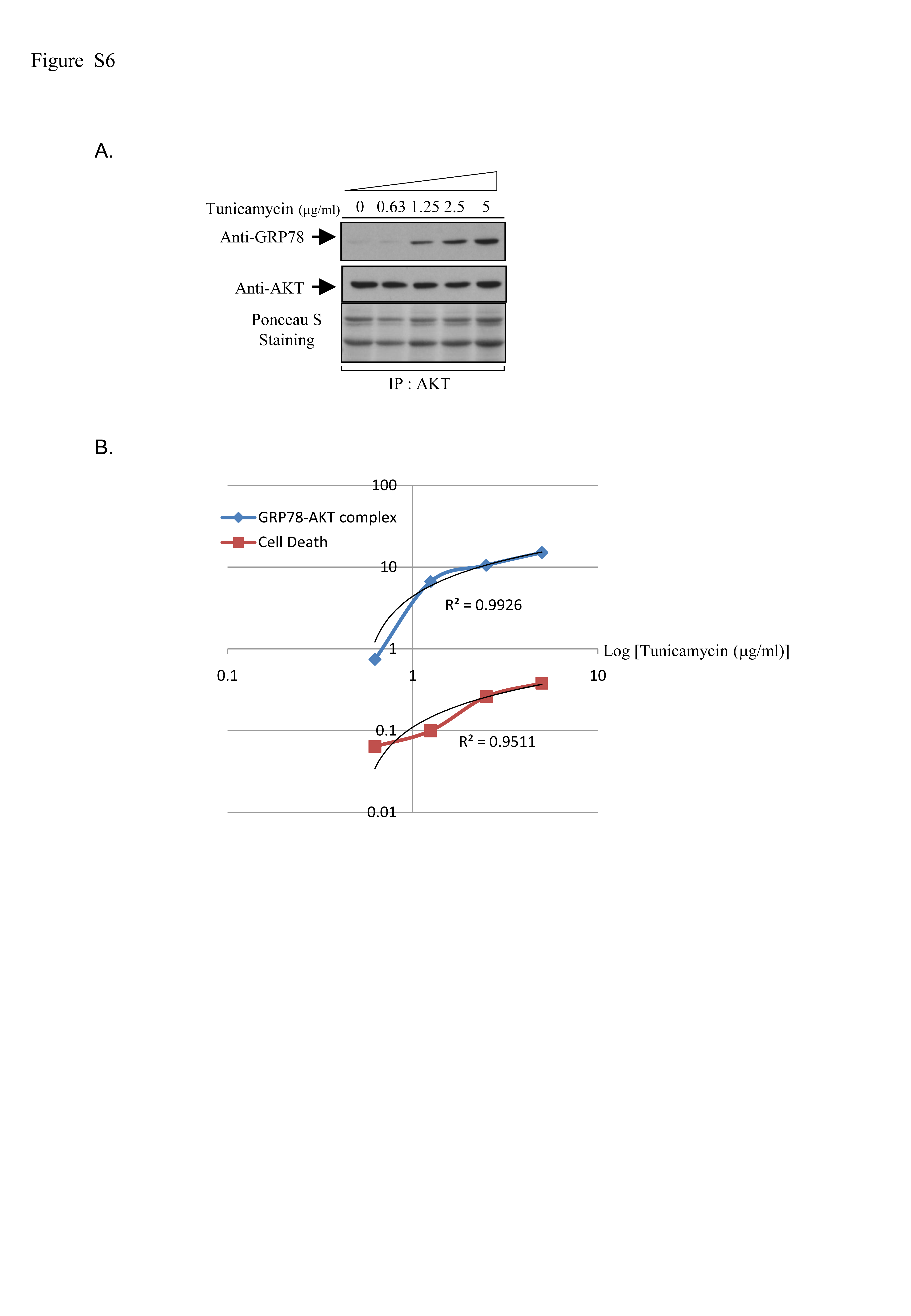

Supplement: Figure S6 — The degree of interaction between GRP78 and AKT is direct proportional to the severity of ER stress. A) A dose-response study of tunicamycin. JEG-3 cells were treated with different concentrations of tunicamycin (0, 0.625, 1.25, 25 and 5 µg/ml) for 24 hour. Proteins were isolated for immunoprecipitation with AKT (1G1) antibody, followed by immunoblotting for GRP78 and AKT. Ponceau S staining was used to show both equivalent input of antibody (IgG heavy chain) and total protein. B) A graph plotted between the amount of AKT-GRP78 immuno-complex obtained from (A) and the percentage of cell death against the concentration of tunicamycin on a Log scale. (TIF) [file pone.0017894.s006.tif]
